# Supplementary material for: CELO Fiber1 Knob Is a Promising Candidate to Modify the Tropism of Adenoviral Vectors
Source: Genes (Basel). 2022 Dec 8;13(12):2316. doi: 10.3390/genes13122316 (PMC9778213; doi:10.3390/genes13122316)
Supplement: Supplementary file 1 [file genes-13-02316-s001.zip › genes-1991382-supplementary.pdf]

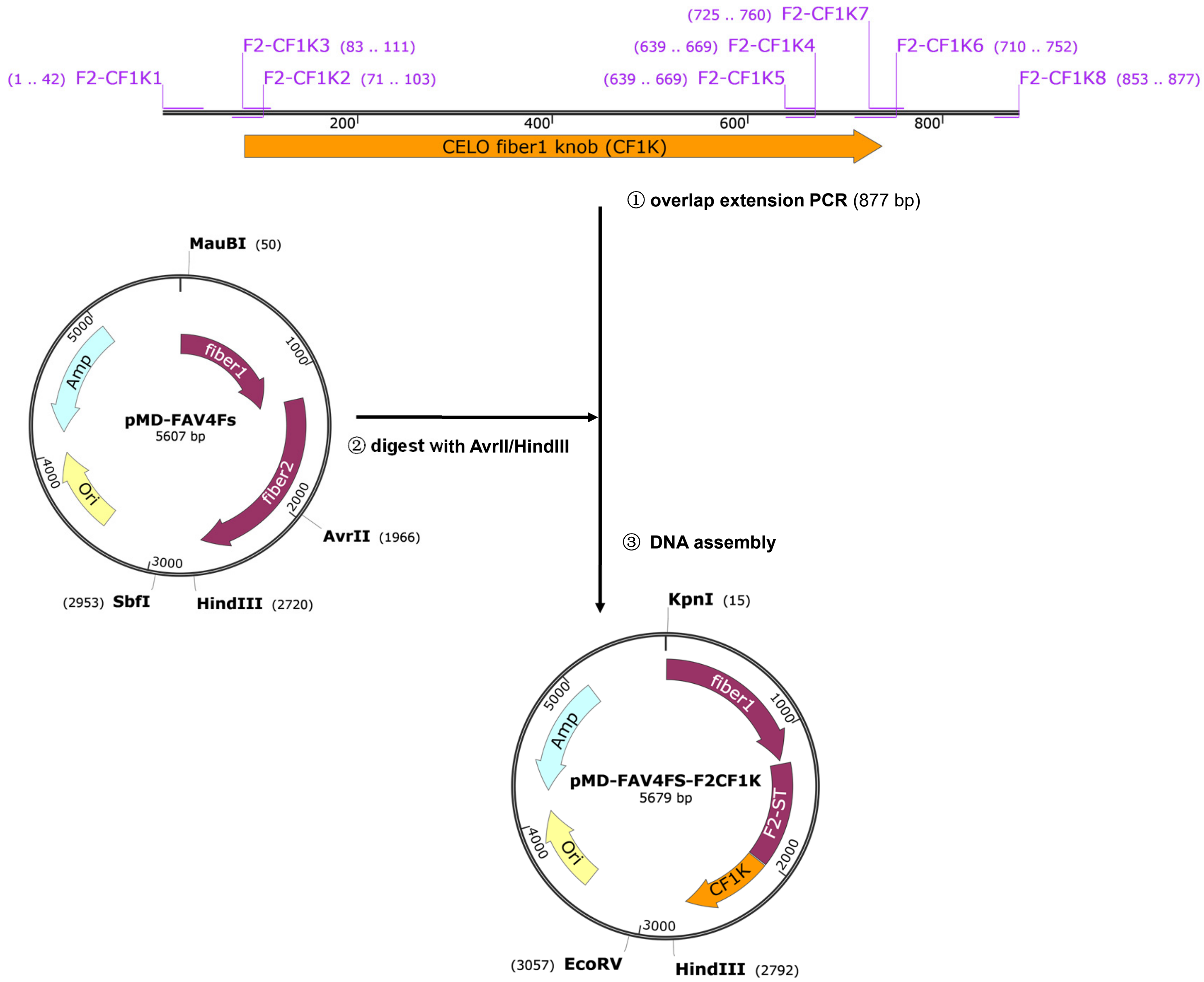

Figure S1. Construction of the intermediate plasmid pMD-FAV4FS-F2CF1K
CF1K: CELO fiber1 knob; F2-ST: FAdV-4 fiber2 shaft and tail.

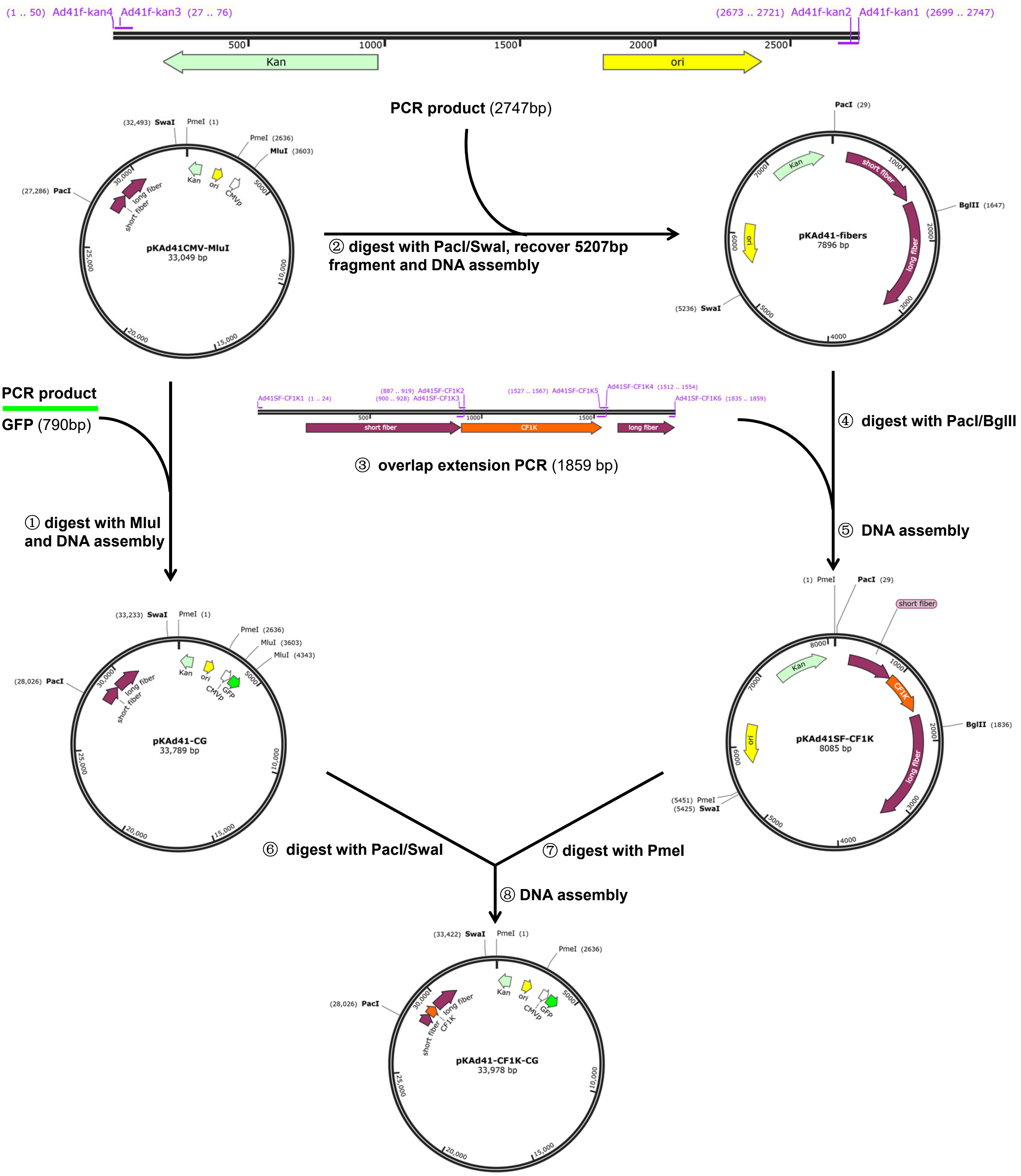

Figure S2. Schematic diagram of construction of recombinant adenoviral plasmid pKAd41-CF1K-CG. CF1K: CELO fiber1 knob

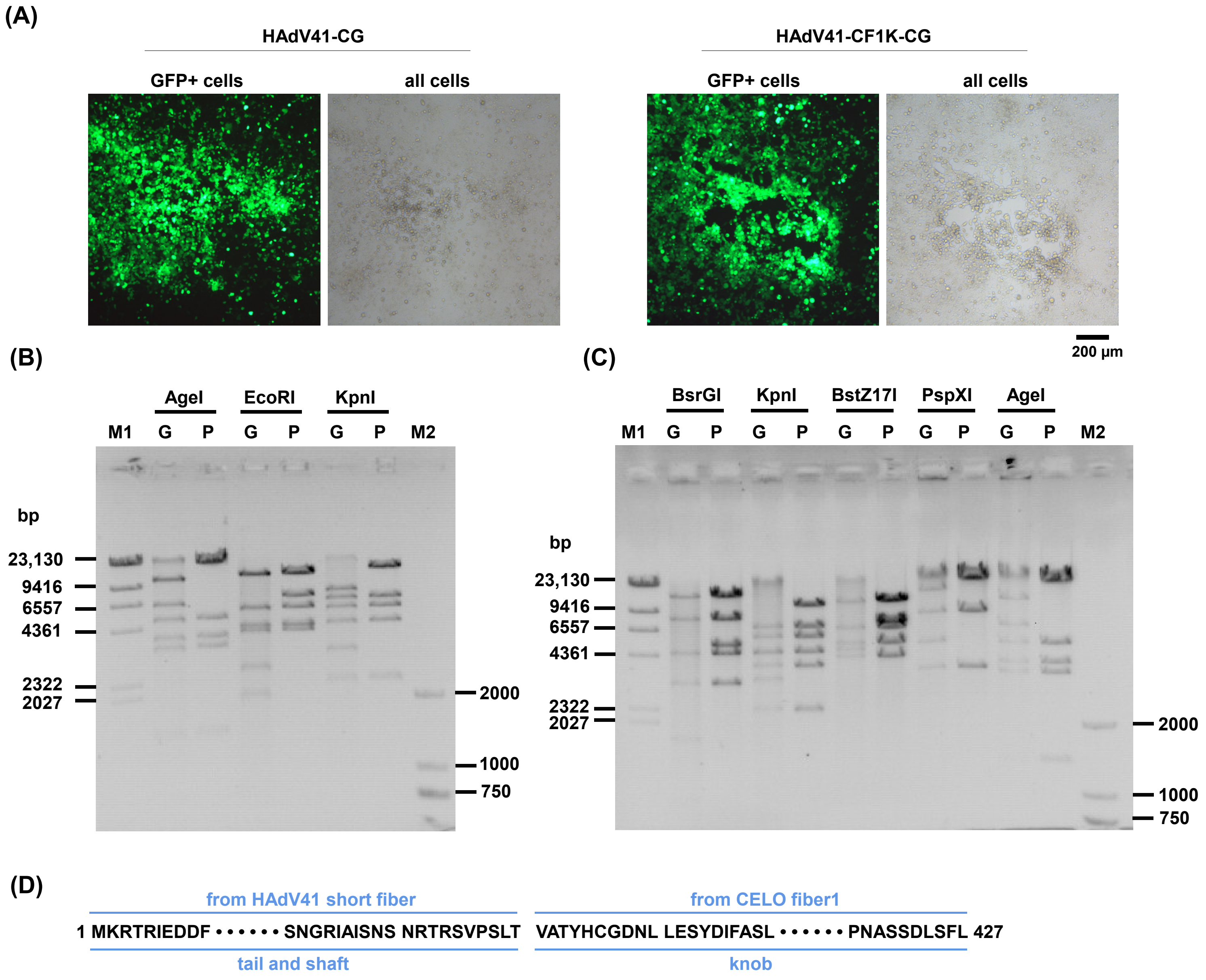

Figure S3. Rescue and identification of fiber-modified HAdV41 vectors.
(A) Rescue of HAdV41-CG and HAdV41-CF1K-CG in 293TE32 cells. (B) The predicted molecular weights (bp) of digested fragments of HAdV41-CG genome were 11,172, 6440, 4959, 3823, 3378, 1382 for AgeI; 12,228, 5807, 4401, 4117, 2634, 1967 for EcoRI; and 8097, 6797, 5732, 4550, 3184, 2284, 510 for KpnI. The predicted molecular weights (bp) of digested fragments of pKAd41-CG plasmid were 20,247, 4959, 3823, 3378, 1382 for AgeI; 12,228, 7236, 5807, 4401, 4117 for EcoRI; and 13,916, 6797, 5732, 4550, 2284, 510 for KpnI. (C) The predicted molecular weights (bp) of digested fragments of HAdV41-CF1K-CG genome were 13,279, 8026, 4504, 3062, 1688, 784 for BsrGI; 6797, 5732, 4550, 4523, 3763, 3184, 2284, 510 for KpnI; 10,850, 6525, 5100, 4673, 4195 for BstZ17I; 13,965, 8341, 5037, 3514, 486 for PspXI and 11,172, 6629, 4959, 3823, 3378, 1382 for AgeI. The predicted molecular weights (bp) of digested fragments of pKAd41-CF1K-CG plasmid were 13,279, 8026, 5107, 4504, 3062 for BsrGI; 10,342, 6797, 5732, 4550, 3763, 2284, 510 for KpnI; 10,850, 7301, 6525, 5100, 4202 for BstZ17I; 21,637, 8341, 3514, 486 for PspXI and 20,436, 4959, 3823, 3378, 1382 for AgeI. (D) Amino acid sequence of the fused fiber protein. M1, lambda/HindIII DNA marker; M2, DL2000 DNA marker.

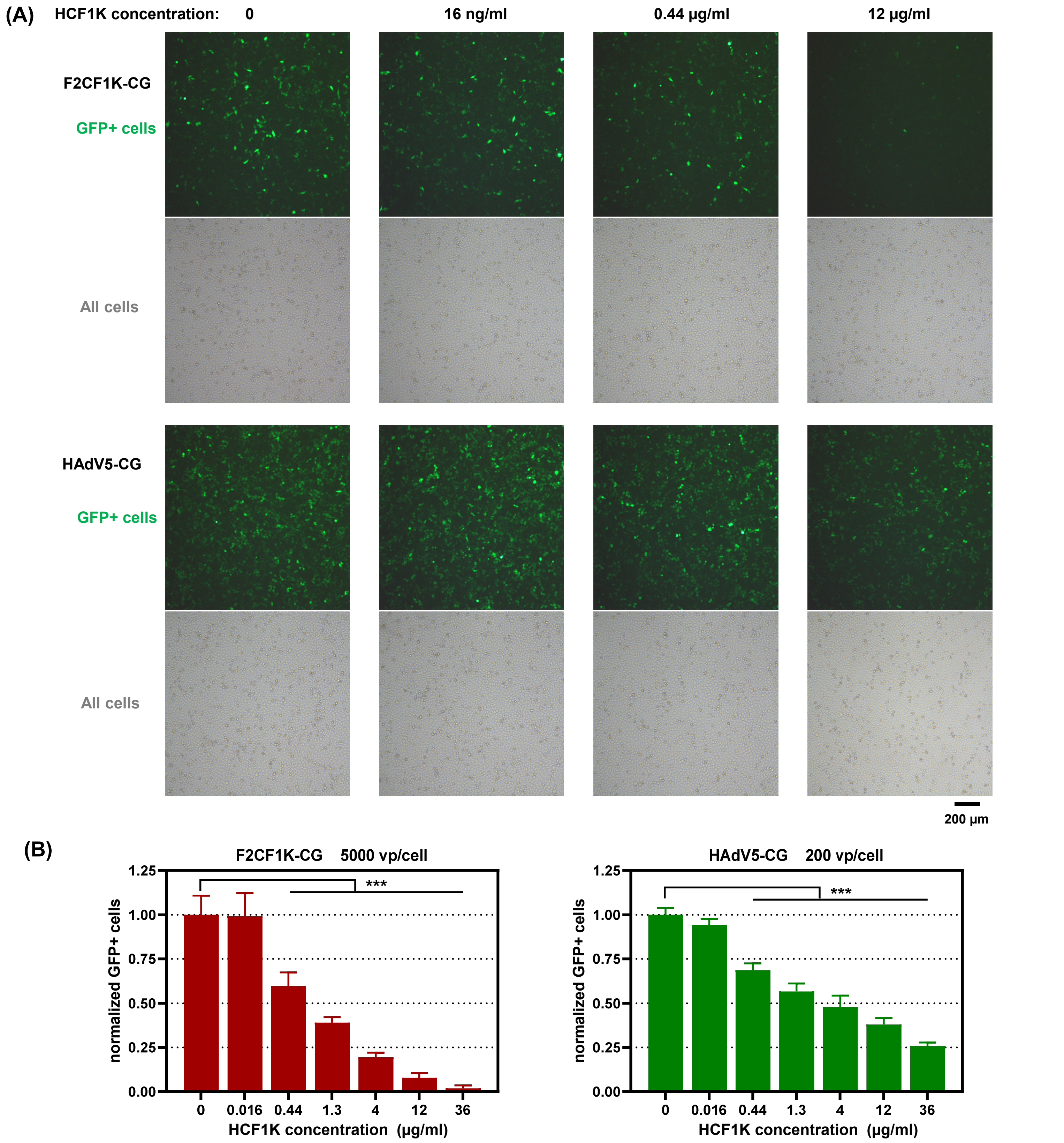

Figure S4. Block the infection of F2CF1K-CG or HAdV5-CG to HEp-2 cells with recombinant CELO fiber1 knob
(A) Recombinant CELO fiber1 knob (HCF1K) was prokaryotically expressed and purified. HEp-2 cells were incubated with HCF1K at indicated concentrations for 30 minutes before infected with F2CF1K-CG at an MOI of 5000 vp per cell or HAdV5-CG virus at an MOI of 200 vp per cell for 2 hours. GFP+ cells were photographed under fluorescence microscope 24 hours post infection.
(B) The intensity of GFP expression in each well was further measured on a multifunctional microplate reader and normalized to that in wells without the addition of HCF1K.
All of the experiments were performed in triplicate and the data shown are from one representative experiment out of the two performed. ***$p < 0.001$
